# Study of the Stability of Structural Variants and Magnetic Properties of TmGa_2_

**DOI:** 10.1021/acs.inorgchem.5c06073

**Published:** 2026-05-22

**Authors:** Sudip Malick, Michał J. Winiarski, Hanna Świątek, Tomasz Klimczuk

**Affiliations:** Faculty of Applied Physics and Mathematics and Advanced Materials Center, 49557 Gdansk University of Technology, Narutowicza 11/12, 80-233 Gdansk, Poland

## Abstract

We present a detailed study of the electronic structure, chemical bonding, and magnetic properties of TmGa_2_. The analysis of chemical pressure in a series of hexagonal REGa_2_ compounds (RE = La, Pr, Nd, Gd–Tm and Lu) reveals that steric factors contribute to the instability of the AlB_2_-type phase of TmGa_2_ and its transition to the orthorhombic KHg_2_-type crystal structure. Magnetic properties have been investigated in single crystals grown using a Pb flux. The temperature-dependent magnetic susceptibility measurements at different crystallographic orientations indicate an antiferromagnetic phase transition at *T*_N_ = 3.5 K. In the low-temperature regime, magnetic susceptibility deviates from the Curie–Weiss law, showcasing a hump attributed to the crystalline electric field (CEF) effect. The CEF effect has been studied thoroughly based on a point charge model of an isostructural compound TmCu_2_, indicating the splitting of 13 nondegenerate levels in TmGa_2_. Isothermal magnetization along *H*∥*c* exhibits a metamagnetic transition at μ_0_ *H*_c_ = 0.15 T. The overall magnetization remains low, with respect to the theoretical value for Tm^3+^, and does not reach saturation even at 9 T and 2 K. Temperature-dependent heat capacity data confirm the magnetic phase transition at *T*_N_ and indicate the presence of the Schottky anomaly. The reduced value of magnetization, the jump in heat capacity, and the magnetic entropy at the magnetic ordering temperature, along with substantial magnetic anisotropy, suggest that CEF interaction plays a crucial role in determining the magnetic ground state in TmGa_2_. The nonmagnetic analogue LuGa_2_ has also been investigated down to 340 mK, but no superconducting transition has been detected. By employing density functional theory calculations, chemical bonding study, and machine learning-based chemical pressure analysis, we were able to explain the instability of the AlB_2_-type structure observed for two of the members of the REGa_2_ (RE = lanthanides and Y) series (TmGa_2_ and LuGa_2_) due to pressure-optimizing transition from the hexagonal AlB_2_-type structure.

## Graphical abstract

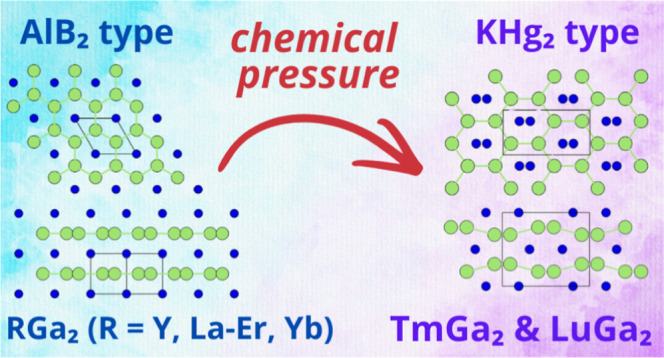

## 1 Introduction

The magnetic ground states of rare-earth (RE) intermetallics are primarily determined by competing Kondo, Ruderman–Kittel–Kasuya–Yosida (RKKY), and crystal electric field interactions. The Kondo interaction (screening of local magnetic moments by conduction electrons in a metallic material) tries to achieve a nonmagnetic ground state with enhanced electron effective mass, whereas RKKY induces magnetic ordering (via spin polarization of conduction electrons by local moments resulting in an oscillatory exchange interaction). The competition between the two may lead to quantum criticality. On the other hand, CEF predominantly splits the spin–orbit coupled multiplets of the 4f electrons, which has a significant impact on the magnetic structure and results in considerable anisotropy.^1–5^ These interactions lead to intriguing physical properties in rare earth-based compounds, including heavy Fermion behavior, the Kondo effect, complex magnetic structures, metamagnetism, valence fluctuation, and large magnetic anisotropy.^6–12^ REGa_2_ family is one of the examples of such rare-earth systems in which the magnetic ground state is mainly governed by the long-range RKKY interaction and CEF effect.^13–20^ The magnetic ordering temperatures and crystal structures of most of the REGa_2_ compounds have been reported a few decades ago, as summarized in Table 1. Almost all of the compounds exhibit a complex antiferromagnetic order within the temperature range of 4–15 K.^13,14^ In certain compounds like CeGa_2_,^15^ TbGa_2_ ^16^ and GdGa_2_,^17^ an incommensurate antiferromagnetic structure is observed. The field-induced multistep magnetic phase transitions are reported in the basal plane in HoGa_2_ and DyGa_2_, resulting in unusual magnetotransport behavior.^18^ It has also been found that PrGa_2_, TbGa_2_, and GdGa_2_ exhibit the anomalous Hall effect, associated with the metamagnetic transition.^21^ REGa_2_ compounds are gaining interest lately due to their hexagonal structure (space group *P*6/*mmm*) in which the alternating layers of Ga and RE atoms form a two-dimensional triangular lattice, which can lead to magnetic frustration.^19,20^ Recently, the magnetic phase diagram has been studied in detail in the triangular lattice GdGa_2_ using resonant elastic X-ray scattering, revealing a cycloidal spin texture. It further suggests that GdGa_2_ may host the Néel-type topological skyrmion lattice phase.^19^ Likewise, a metamagnetic multiband Hall effect and Ising-type antiferromagnetism are observed in ErGa_2_.^20^ The nonmagnetic compounds YGa_2_ and LaGa_2_ were subsequently reported to exhibit superconductivity at temperatures below 1.2 K.^22,23^

**Table 1:** Crystal Structure and Magnetic Ordering Temperature of Rare-Earth Gallides REGa_2_ at Ambient Pressure

RE	crystal structure	*T*_N_ (K)	reference:
Y	hexagonal	superconductor	^23^
La	hexagonal	superconductor	^22^
Ce	hexagonal	4.1	^13^
Pr	hexagonal	7.3	^13^
Nd	hexagonal	9.2	^13^
Gd	hexagonal	12.1	^13^
Tb	hexagonal	14.8	^13^
Dy	hexagonal	6.4	^13^
Ho	hexagonal	8.0	^13^
Er	hexagonal	7.5	^13^
**Tm**	orthorhombic	3.5	[this study]
Yb	hexagonal	nonmagnetic	^26^
Lu	orthorhombic	nonmagnetic	[this study]

Interestingly, TmGa_2_ and LuGa_2_ are the only two compounds within this family that adopt a KHg_2_-type orthorhombic structure (space group *Imma*) at ambient pressure. At high pressure (∼21 GPa), TmGa_2_ changes its structure from orthorhombic to an UHg_2_-type hexagonal structure.^24^ As discussed by Hoffmann and Pöttgen,^25^ the AlB_2_-, UHg_2_- and KHg_2_-type structures are closely related. The latter can be viewed as a variant of the former two with a broken 6-fold symmetry. The AlB_2_ and KHg_2_ structure types are compared in Figure 1. The key differences between the crystal structures of AlB_2_-type REGa_2_ (RE = Y, La–Er, Yb) and TmGa_2_/LuGa_2_ are (1) broken hexagonal symmetry and (2) interlayer bonding.

**Figure 1:**
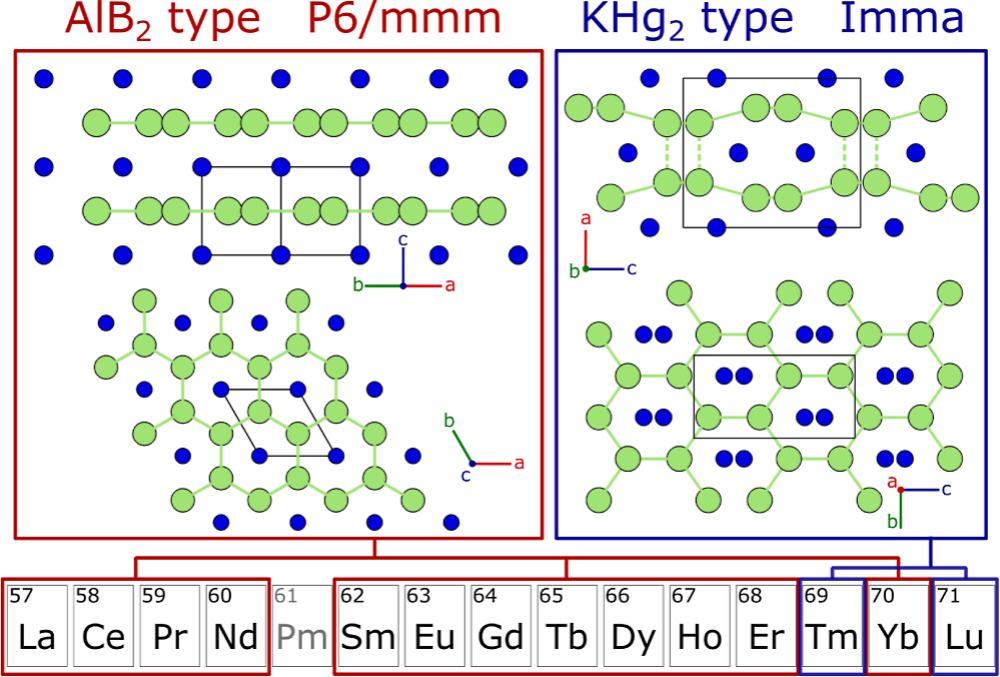
Comparison of the hexagonal AlB_2_-type and the orthorhombic KHg_2_-type structure. Note that in TmGa_2_ and LuGa_2_ crystallizing in the latter, the interlayer distance is comparable with the Ga–Ga spacing within the honeycomb-like layers, resulting in an effectively 4-connected network, compared to the 3-connected one found in the AlB_2_-type structure. Ga and RE are represented by the green and blue symbols, respectively.

The ground-state properties of orthorhombic TmGa_2_ have not been reported to date. Here, we have grown single crystals of TmGa_2_ and its nonmagnetic analogue, LuGa_2_, and performed a systematic study of its magnetic properties using magnetization and heat capacity measurements. Our results reveal that TmGa_2_ undergoes an antiferromagnetic phase transition around 4 K in the presence of CEF. The magnetic and thermodynamic properties of TmGa_2_ are significantly influenced by the CEF effect. We also performed ab initio calculations of the electronic structure and chemical bonding in order to understand the reason for the preference of the KHg_2_-type orthorhombic structure in TmGa_2_ and LuGa_2_.

## 2 Materials and Methods

Single crystals of TmGa_2_ and LuGa_2_ were grown using Pb flux. High-purity elements Pb (Onyxmet, 99.99%), Ga (Onyxmet, 99.99%), and either Tm (Onyxmet, 99.9%) or Lu (Onyxmet, 99.9%) were taken in a 10:2:1 molar ratio. The elements were then placed in an alumina crucible and sealed in a quartz ampule under partial argon pressure. The whole assembly was then heated to 1050 °C and soaked for 20 h in a chamber furnace. The furnace was slowly cooled at a rate of 2 °C/h, followed by centrifuging at 700 °C to remove the Pb flux. The crystals are typically rod-like with a length of a few millimeters, as shown in Figure 2b. Before investigating the physical properties, the crystals were further etched to remove Pb from their surfaces using a mixture of acetic acid and H_2_O_2_ in equal proportions.

**Figure 2:**
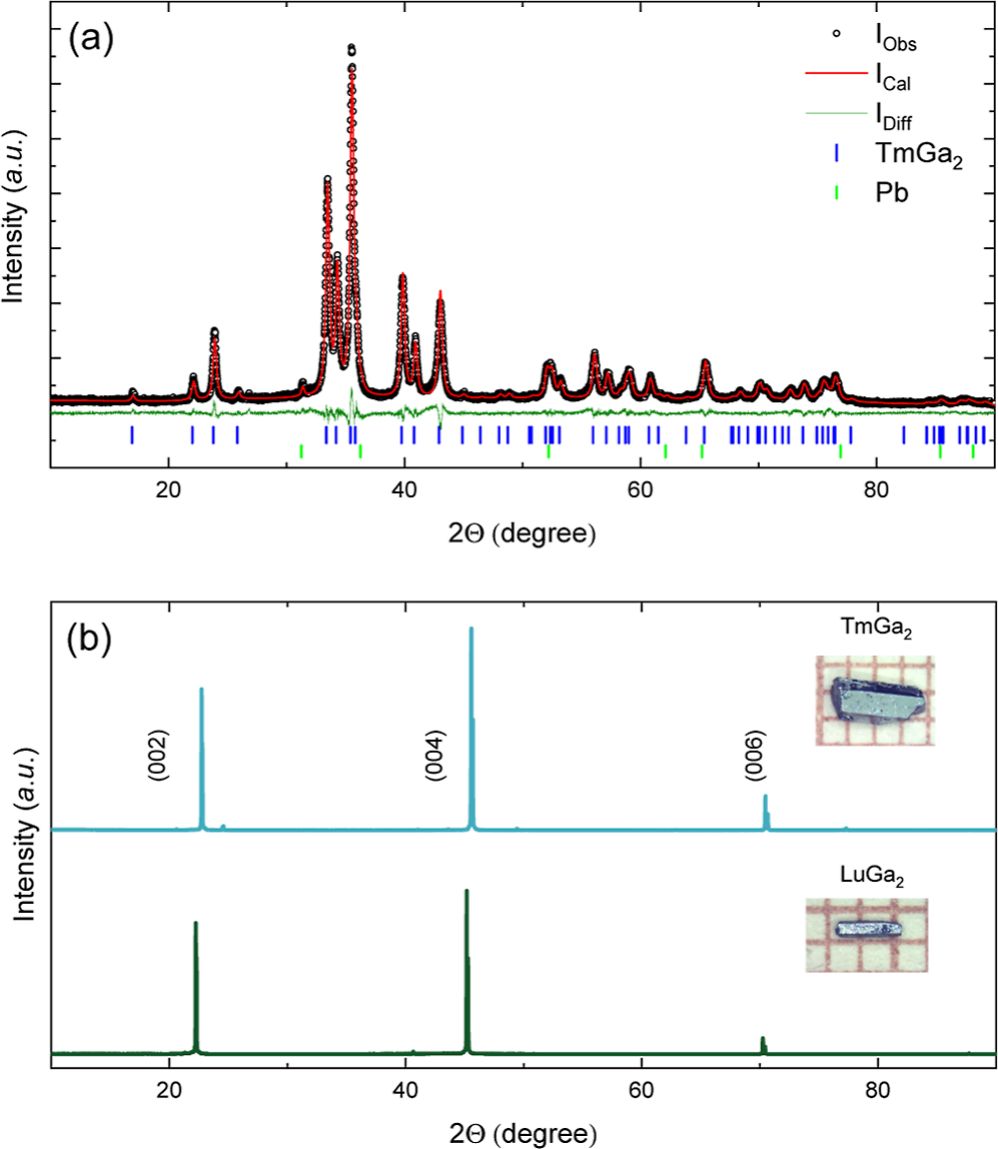
(a) The powder X-ray diffraction (XRD) pattern of the crushed TmGa_2_ single crystals recorded at room temperature, shown as black dots. The calculated pattern (Rietveld) is shown by the red line. The Bragg positions are represented by the blue (TmGa_2_) and lime (Pb) vertical ticks. The difference between the observed intensity and the estimated intensity is represented by the green line. (b) XRD pattern taken using a powder diffractometer on single crystals of TmGa_2_ and LuGa_2_. The optical pictures of the crystals are displayed in the attached image.

XRD was carried out to confirm the crystal structure and crystal orientations in a Bruker D2 Phaser diffractometer equipped with a Cu lamp and a LynxEye XE-T position-sensitive detector. The chemical composition of the obtained crystals was checked using energy-dispersive X-ray spectroscopy (EDS) in an FEI Quanta FEG 250 electron microscope. The EDS spectra were analyzed by means of the standardless quantification method, as implemented in EDAX TEAM software. Magnetic measurements of the compound were performed using a vibrating sample magnetometer attached to the Quantum Design EverCool-II physical property measurement system (PPMS). Measurements of heat capacity were conducted using the two-τ relaxation method on the PPMS platform.

Density functional theory (DFT) calculations of the electronic structure of the orthorhombic and hexagonal variants of TmGa_2_ were performed using the Quantum Espresso (QE) ver. 7.2 package,^27–29^ employing the projector-augmented wave (PAW) method^30^ and the Perdew–Burke–Ernzerhof Generalized Gradient Approximation (PBE GGA) of the exchange–correlation (xc) functional.^31^ PAW sets for Ga and Tm (4f-in-core configuration) were taken from the PSLibrary (Ga.pbe-dn-kjpaw_psl.1.0.0.UPF, Tm.pbe-spdn-kjpaw_psl.1.0.0.UPF).^32^ In the self-consistent field calculations, the *k*-point mesh was set to 9 × 9 × 9 and the wave function and charge density cutoffs to 65 and 650 Ry, respectively. Unit cell parameters and (in case of the orthorhombic variant only) atomic positions were relaxed using the BFGS method. For comparison, the same method was used to relax the crystal structure of ErGa_2_ and HoGa_2_ (see Tables S2 and S3 in the Supporting Information). Projected Crystal Orbital Hamilton Population and Crystal Orbital Bond Index (COBI) analysis were performed using the LOBSTER 5.1.1 code^33–35^ by projecting the results of QE plane-wave calculations (calculated in a non-SCF scheme with *k*-point grid density increased to 13 × 13 × 13) to the local-orbital basis pbeVASPfit2015.^33^ Chemical pressure analysis was performed using the machine learning-based ML-CP model by van Buskirk, Peterson, and Fredrickson.^36^ To maintain consistency with the xc functional used in ML-CP training set, unit cells of AlB_2_-type REGa_2_ (RE = Y, La, Pr, Nd, Gd–Tm, Lu) were relaxed using QE with the PAW method and the Perdew–Zunger Local Density Approximation (PZ LDA)^37^ (with 4f states treated as core states). The corresponding PAW data sets were taken from the PSLibrary.^32^ The resulting unit cell parameters are shown in Table S4 in the Supporting Information. Additionally, we performed the structural relaxation of YGa_2_ with Hartwigesen-Goedecker-Hutter (HGH) PZ LDA pseudopotentials (Y.pz-hgh.UPF, Ga.pz-hgh.UPF),^38^ which were used in ML training data set^36,39^ (Table S5 in the Supporting Information). The *k*-point mesh size and cutoffs were kept the same as in PAW calculations. The results are presented in Table S5 in the Supporting Information. The input Crystallographic Information Files were created using the FINDSYM program of the ISOTROPY suite.^40^ Crystal structure images were rendered using the VESTA v. 3.4.5 program.^41^ Crystal electric field (CEF) calculations were performed using the PyCrystalField v. 2.3.11 package.^42^

## 3 Results and Discussions

### 3.1 Structural Stability

Figure 2a presents the powder XRD pattern of crushed single crystals of TmGa_2_. The XRD data have been analyzed utilizing the Rietveld refinement method^43^ in the Bruker TOPAS software, indicating that TmGa_2_ crystallizes in a KHg_2_-type orthorhombic structure with space group *Imma* (No. 74). A schematic diagram of the crystal structure is given in Figure 1. The refined lattice parameters are *a* = 4.2121(2) Å, *b* = 6.8924(3) Å, and *c* = 8.0679(3) Å, consistent with an earlier report.^44^ Other XRD refinement parameters for TmGa_2_ and LuGa_2_ are provided in the Supporting Information. Figure 2b shows the XRD patterns taken on single crystals of TmGa_2_ and LuGa_2_, exhibiting sharp reflections along the (00*l*) plane. This observation indicates that the crystallographic *c*-axis is oriented perpendicular to the flat plane of the single crystals. The pronounced peaks also signify the high crystalline quality of the grown single crystals. The EDS data [see Figure S2] from the as-grown crystals of TmGa_2_ and LuGa_2_ show the expected 1:2 ratio, with a maximum error of 3%.

In the relaxed hexagonal variant of TmGa_2_ (*a* = 4.18146 Å, *c* = 4.03587 Å) the intralayer Ga–Ga bond length is 2.414 Å and the interlayer spacing is 4.036 Å, resulting in negligible Ga–Ga orbital overlap along the crystallographic *c* direction as demonstrated in Figure 3a. The ratio between the unit cell parameters *c*/*a* = 0.965 is lower than the closest-packing value *c*/*a* = 1.074, suggesting that the atomic packing along the basal plane is loose and the Ga–Ga honeycomb network is under compression.^45^ For a slightly larger lanthanide atom like Er the relaxed parameters (*a* = 4.18853 Å, *c* = 4.05831 Å) give *c*/*a* = 0.969, and for Ho (*a* = 4.19600 Å, *c* = 4.07806 Å) give *c*/*a* = 0.972 (see Figure 3f). This indicates that the lanthanide contraction effect leads to an increase in compressive strain with increasing atomic numbers in the 4f block.

**Figure 3:**
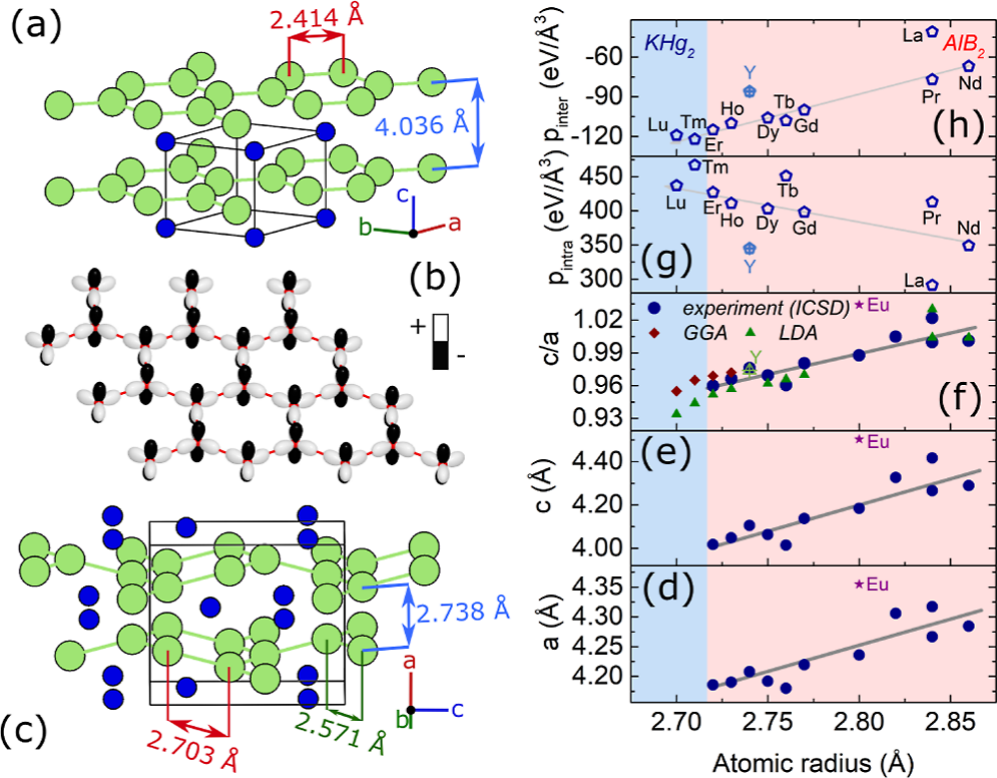
(a) Relaxed crystal structure of the AlB_2_-type TmGa_2_ with Ga–Ga in-plane and out-of-plane distances displayed; RE is labeled as blue and Ga as green. (b) Visualization of the chemical pressure^46^ acting on Ga hexagonal sublattice in AlB_2_-type TmGa_2_—white lobes indicate a compressive stress, black—tensile; (c) relaxed crystal structure of the KHg_2_-type TmGa_2_ with Ga–Ga distances displayed; (d,e) reported unit cell parameters of REGa_2_ (RE = lanthanides) AlB_2_-type compounds (based on the crystal structures reported in the ICSD database); (f) *c*/*a* unit cell parameter ratio for experimentally reported (blue circles) and calculated (using PBE GGA xc functional—red diamonds, and PZ LDA—green triangles). Panels (g) and (h) show the chemical pressure values along the Ga–Ga bonds within honeycomb layers and between them, respectively. Atomic radii are given after Rahm, Hoffmann, and Ashcroft.^47^ Data points for YGa_2_ were plotted using a different color and point style to highlight their deviation from the trend observed for the lanthanide series, which was also observed in the isostructural R_2_PdGe_3_ family.^48^

In contrast with the short intralayer Ga–Ga distances in the hexagonal variant, the relaxed orthorhombic KHg_2_-type TmGa_2_ crystal structure shows in-plane bonding lengths of 2.571 Å and 2.703 Å and a comparable interlayer bond length of 2.738 Å. Thus, the Ga network in the orthorhombic TmGa_2_ shows a three-dimensional character with a 4-connected bonding topology (compared to the 3-connected flat hexagonal layer in the AlB_2_-type variant). The calculated total energy for the relaxed orthorhombic variant is lower by 9.4 meV/atom compared to the hexagonal one, in agreement with the observed stability of the KHg_2_-type structure of TmGa_2_.

Chemical pressure analysis performed using ML-CP shows that in a series of AlB_2_-type REGa_2_ compounds (RE = Gd–Tm, Lu), the pressure component along the in-plane Ga–Ga bond increases with decreasing radius of the atom, with a deviation from the trend seen for RE = Tb and Lu (Figure 3g). Notably, a similar deviation from trend for RE = Tb is also seen in experimental unit cell parameters (Figure 3d–f). The decreasing size of the RE atom also results in a stronger tensile stress between the hexagonal Ga layers, as shown in Figure 3h.

Increasing chemical pressure acting on Ga–Ga bonds results in a distortion of the AlB_2_-type structure to the orthorhombic KHg_2_ for the smallest lanthanide atoms, Tm and Lu. A graphical representation of the calculated chemical pressure is shown in Figure 3b for the hexagonal TmGa_2_. It is worth noting that in this structure the in-plane Ga–Ga bonds are compressed (as shown by white lobes), while the layers seem to be pulled apart (black lobes in the *c* direction). In the relaxed orthorhombic crystal structure of TmGa_2_ the “in-plane” Ga–Ga contacts are significantly longer, while interlayer spacing becomes much shorter (Figure 3a,c). The AlB_2_ to KHg_2_ type transformation is consistent with the relaxation of the chemical pressure. This highlights the steric origins of the instability of the hexagonal variant of TmGa_2_ at ambient pressure. At high pressures (above ca. 20 GPa) TmGa_2_ is reported to form in the UHg_2_ structure type, which is isopointal to AlB_2_, but with *c*/*a* ratio significantly lower than 1. In UHg_2_-type TmGa_2_, the inter- and intralayer bond lengths are comparable in length (*c*/*a* evolves from 0.785 to 0.765 with pressure increasing from 20 to 40 GPa) and the hexagonal symmetry is preserved likely due to higher packing ratio than in the orthorhombic KHg_2_-type.

For validation, we compared the results obtained with ML CP for YGa_2_ unit cells relaxed using PAW and HGH methods (using PZ LDA xc functional in both cases) with the results of DFT-based chemical pressure analysis available in the Intermetallic Reactivity Database (IRD)^39^ (Figure S5 in the Supporting Information). Taking into account the differences in the relaxed unit cell parameters, resulting from differences in the calculation detail and software used, a good qualitative agreement of the Ga–Ga in-plane pressure was found in all three models. The ML predicted CP out-of-plane component was found to show the same sign but a significantly larger magnitude than in the IRD data, which likely results from a noticeably larger *c*/*a* ratio found in our calculations (see Table S5 in the Supporting Information).

Results of DFT calculations on both structural variants of TmGa_2_ are shown in Figure 4a,b,e,f. As expected, the band structure of the hexagonal variant is similar to the one found in other isoelectronic REGa_2_ compounds and the isostructural series Y_2_TGe_3_ (T = Ni, Pd, Pt),^23,49^ with a prominent symmetry-protected band crossing at the *K* point, just above the Fermi level. In the band structure of the orthorhombic variant, a symmetry-protected band crossing is seen just below the Fermi level at the R and W points. It is worth noting that in the Materiae database^50^ the KHg_2_-type TmGa_2_ (id: MAT00013991) is classified as a high-symmetry-point topological semimetal (in the paramagnetic state).

**Figure 4:**
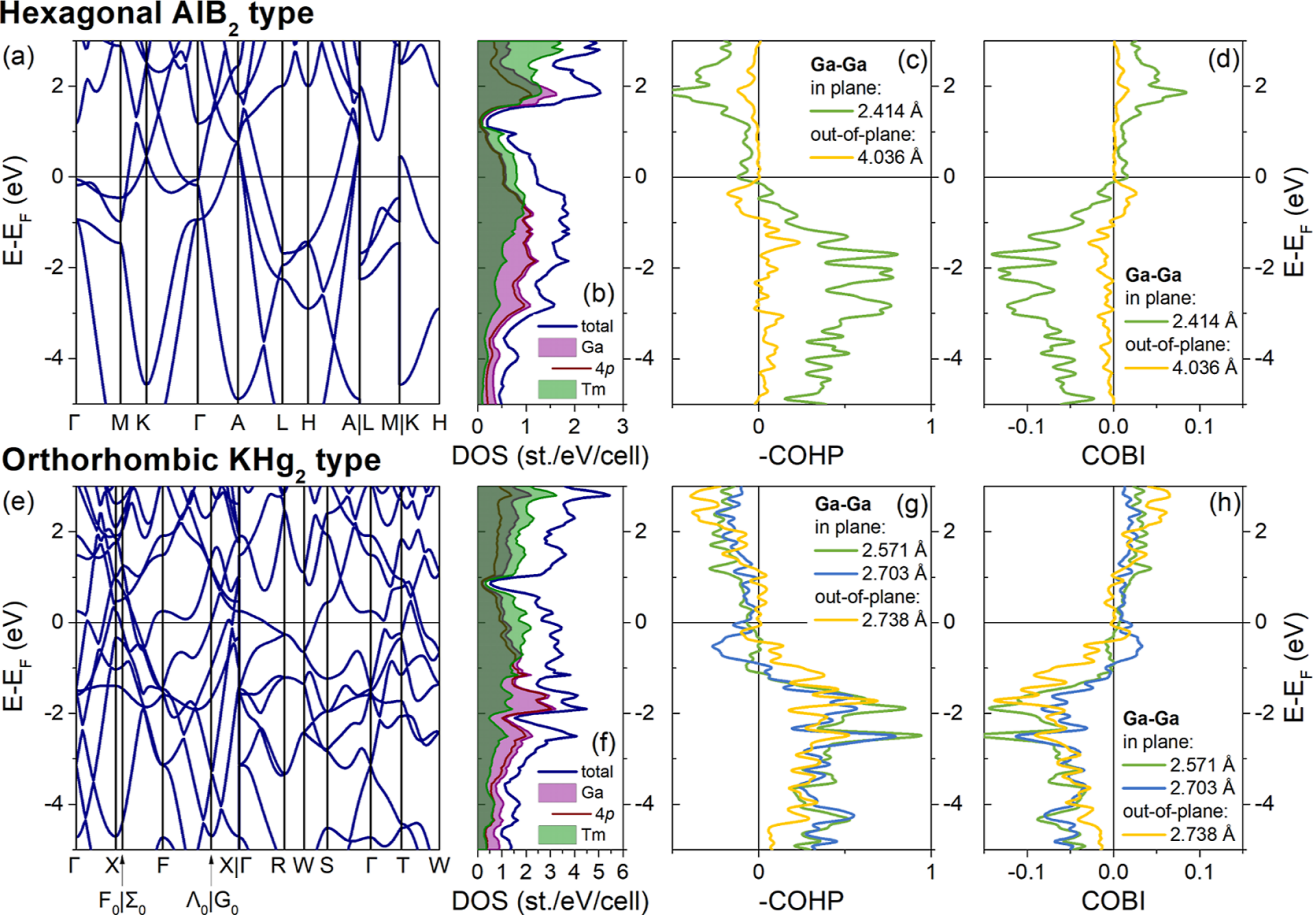
Band structures (a,e), density of states (b,f), COHP (c,g), and COBI (d,h) calculated for the hexagonal and orthorhombic variants of TmGa_2_, respectively.

COHP (Figure 4c,g) and COBI (Figure 4d,h) show that while in the AlB_2_ variant the out-of-plane Ga–Ga are weak and at the Fermi level have an antibonding/nonbonding character, in the KHg_2_ variant in- and out-of-plane interactions are of comparable strength.

### 3.2 Magnetic Properties

Figure 5a illustrates the temperature dependence of the magnetic susceptibility (χ = *M*/*H*) of single-crystalline TmGa_2_ in the temperature range 2–80 K at magnetic field 0.1 T along *H*∥*c* and *H*⊥*c*, respectively. The measurements have been performed for zero-field-cooled (ZFC) and field-cooled (FC) configurations; however, no variations of magnetic susceptibility data have been observed among these configurations. A sharp peak and a kink are observed around 3.5 K along *H*∥*c* and *H*⊥*c*, respectively. These anomalies in the temperature-dependent magnetic susceptibility data suggest an antiferromagnetic phase transition in TmGa_2_. The Neel temperature *T*_N_ = 3.5 K has been estimated as the peak of the dχ­(*T*)/d*T* data. Below the phase transition temperature, a significant drop along *H*∥*c* and a slight upturn along *H*⊥*c* are observed in the susceptibility data. The amplitude of magnetic susceptibility at the ordering temperature is about 5.2 times larger along *H*∥*c* with respect to *H*⊥*c*, which suggests a large magnetic anisotropy in this compound. The inset of Figure 5a shows the inverse magnetic susceptibility measured at 0.1 T in the temperature range 2–300 K. The high-temperature magnetic susceptibility data above 50 K can be expressed well by the modified Curie–Weiss formula

1 $$\chi \left(T\right)=\chi_{0}+\frac{C}{T-\theta_{P}}$$

where Curie constant *C* is directly related to the effective magnetic moment (μ_eff_) through the expression *C* = μ_0_ *μ*_eff_^2^ *N*/3*k*_B_, where μ_0_, *N*, *k*_B_ are known as the magnetic permeability of free space, the number of magnetic atoms per unit volume, and the Boltzmann constant, respectively. The parameters χ_0_ and θ_P_ are the temperature-independent magnetic susceptibility and paramagnetic Curie temperature, respectively. The fitting of the inverse susceptibility data with the CW formula, as shown in the inset of Figure 5a, yields the effective magnetic moment 7.76 μ_B_ and 7.81 μ_B_ for *H*∥*c* and *H*⊥*c*, respectively. The obtained effective moments are close to the theoretical value of Tm^3+^ (7.57 μ_B_). The Curie temperature is found to be 5.9 K and −15.8 K *H*∥*c* and *H*⊥*c*, respectively. The positive and negative value of θ_P_ might indicate that the magnetic interaction in the paramagnetic state show ferromagnetic and antiferromagnetic correlations along *H*∥*c* and *H*⊥*c*, respectively. On the other hand, as noted by Mugiraneza & Hallas,^51^ for lanthanide ions with *J* ≠ 0, the value of θ_P_ must be treated with caution due to the contribution from thermally populated excited states, resulting in overestimated interaction strength. Moreover, correlation between fitting parameters θ_P_ and χ_0_ can introduce systematic errors in estimation of both. In order to fully clarify the sign and strength of exchange interactions ab initio calculations and/or neutron diffraction and scattering experiments would be necessary.

**Figure 5:**
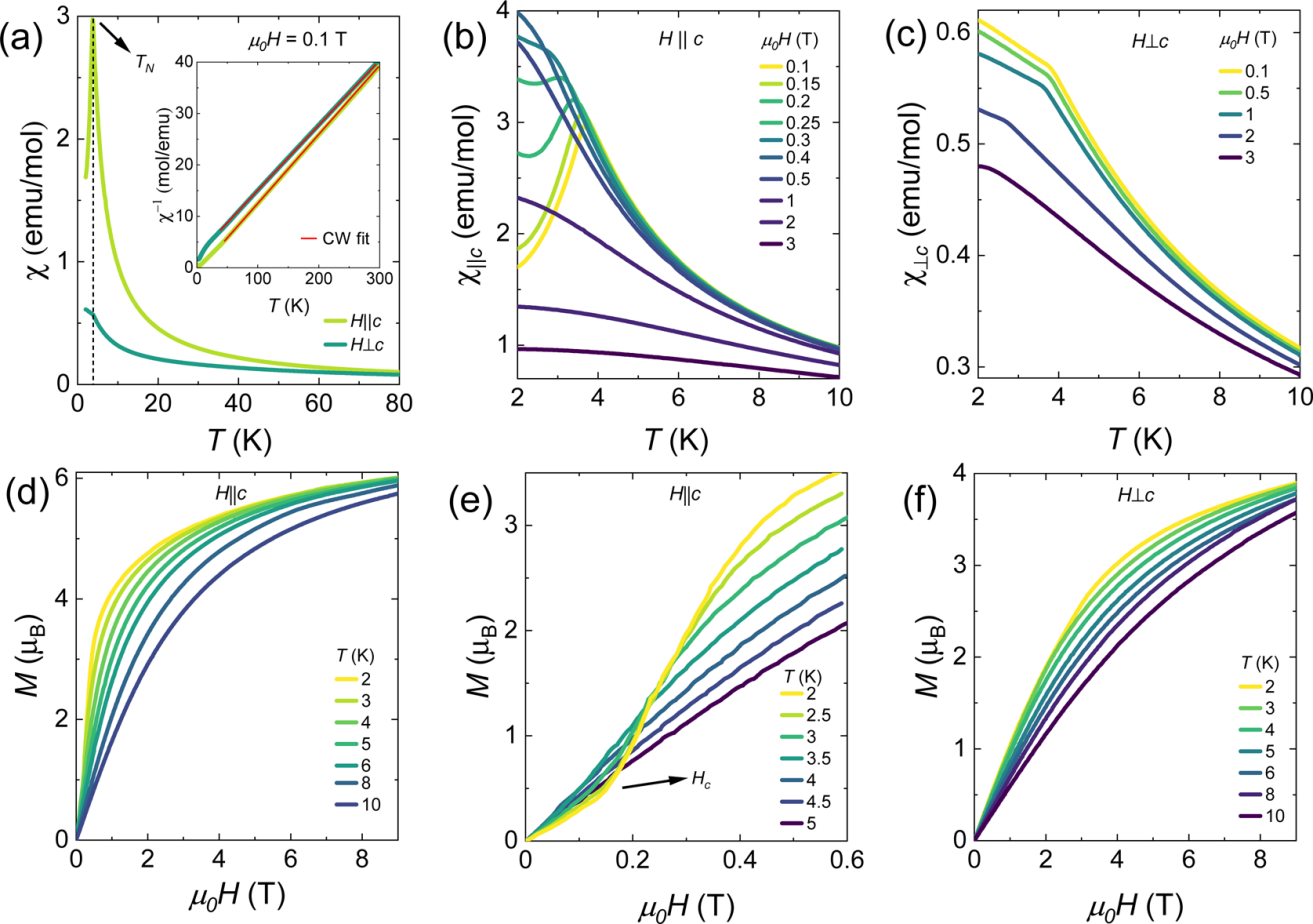
(a) The temperature-dependent magnetic susceptibility measured at 0.1 T for *H*∥*c* and *H*⊥*c*. The inset shows the inverse susceptibility as a function of temperature for different crystallographic orientations in the temperature range 2–300 K. Panels (b) and (c) present the effects of magnetic field strength on the magnetic ordering temperature in the temperature-dependent susceptibility data for *H*∥*c* and *H*⊥*c*, respectively. (d) and (f) show the field dependence of magnetization collected at various fixed temperatures for *H*∥*c* and *H*⊥*c*, respectively. (e) Magnetization within the low field regime, which reveals metamagnetic phase transition along *H*∥*c*.

Below 50 K, the inverse susceptibility data deviate from the CW law, and a broad hump is observed around 15 K along *H*⊥*c*, which is a typical signature of the crystal electric field effect. Such temperature dependence has been observed in several rare-earth compounds, including Tm-based compounds like TmPtIn^12^ and TmNiC_2_.^52^ Figure 5b,c shows the magnetic susceptibility data measured at various applied fields along two crystallographic orientations. The anomaly shifts toward a lower temperature and becomes less pronounced as the applied magnetic field increases, which is a typical signature of the AFM phase transition.^53^

The field-dependent magnetization data acquired along *H*∥*c* and *H*⊥*c* for various fixed temperatures up to 9 T are shown in Figure 5d,f, respectively. Isothermal magnetization at 2 K shows linear field dependence at low field regime as expected for an AFM system; however, a jump in the magnetization above μ_0_ *H*_c_ = 0.15 T is observed for *H*∥*c* as clearly visible in Figure 5e. This jump in magnetization is possibly due to field-induced metamagnetic transition. As the temperature increases, the critical field *H*_c_ moves to a lower field and disappears above 4 K. The magnetization does not show any saturation tendency up to 9 T. The maximum value of magnetization at 2 K reaches 6 μ_B_ and 4 μ_B_ at 9 T along *H*∥*c* and *H*⊥*c*, respectively. These values are much less than the expected value of saturation magnetization of 7 μ_B_ for Tm^3+^, which suggests the presence of strong crystalline electric field interactions in TmGa_2_. A similar value of saturation magnetization is observed in several Tm-based intermetallic compounds.^12,52,54^

Next, a CEF model for TmGa_2_ was constructed by adjusting the parameters calculated for the isostructural TmCu_2_ ^55,56^ to match the measured magnetic heat capacity and temperature-dependent susceptibility. TmGa_2_ and TmCu_2_ crystallize in an orthorhombic structure with point symmetry *D*_2*h*_, thus the CEF Hamiltonian can be defined as

2 $$H_{CEF}=\sum_{l\in \left\{2,4,6\right\}}\sum_{\begin{array}{l} m=0\\ m\to even \end{array}}^{l}B_{l}^{m}O_{l}^{m}$$

where *O*_*l*_^*m*^ are the Stevens operators which are functions of angular momentum *J*. *B*_*l*_^*m*^ are the CEF parameters.^57,58^ The temperature-dependent CEF susceptibility is expressed as follows

3 $$\begin{array}{l} \chi_{CEF}^{i}=\frac{N_{A}{\left(g_{J}\mu_{B}\right)}^{2}}{Z}[\sum_{n}\beta |\langle n|J_{i}|n\rangle {|}^{2}e^{-\beta E_{n}}\\ +\sum_{n\neq m}|\langle m|J_{i}|n\rangle {|}^{2}\frac{e^{-\beta E_{n}}-e^{-\beta E_{m}}}{E_{m}-E_{n}}] \end{array}$$

where *N*_A_ and *g*_J_ are the Avogadro constant and Landé factor, respectively. *J*_i_ is the component of angular momentum, $Z=\sum_{n}e^{-\beta E_{n}}$ , where β = 1/*k*_B_ *T*. |*n*⟩ is the *n*th eigenfunction with eigenvalue *E*_*n*_.^59^ The first term of the above equation is the Curie contribution to the susceptibility, and the second is the Van Vleck susceptibility. The magnetic susceptibility can be further redefined considering molecular field (λ_*i*_) as

4 $${\left(\chi_{i}-\chi_{0}\right)}^{-1}={\left(\chi_{CEF}^{i}\right)}^{-1}-\lambda_{i}$$

The calculated temperature-dependent inverse magnetic susceptibilities for various crystallographic orientations agree reasonably with the overall experimental data, as displayed in Figure 6a. We should note that the CEF analysis is based on a simplified single-ion model that neglects interactions such as anisotropic exchange, magnetoelastic coupling, etc.;^60–62^ as a result, slight deviation from the theoretical model is observed. Since *J* = 6 for Tm^3+^, the total multiplet (2*J* + 1) = 13 for orthorhombic point symmetry (*D*_2*h*_) splits into 13 singlets.^63^ The estimated CEF parameters and corresponding energy levels and wave functions are presented in Table 2. A schematic diagram of energy level splitting is presented in Figure 6b. The CEF parameter *B*_2_^0^ is also estimated from the Curie–Weiss temperatures for different crystal orientations using the formula^64^

5 $$\theta_{P}^{ab}-\theta_{P}^{c}=\frac{3}{10}B_{2}^{0}\left(2J-1\right)\left(2J+3\right)$$

**Figure 6:**
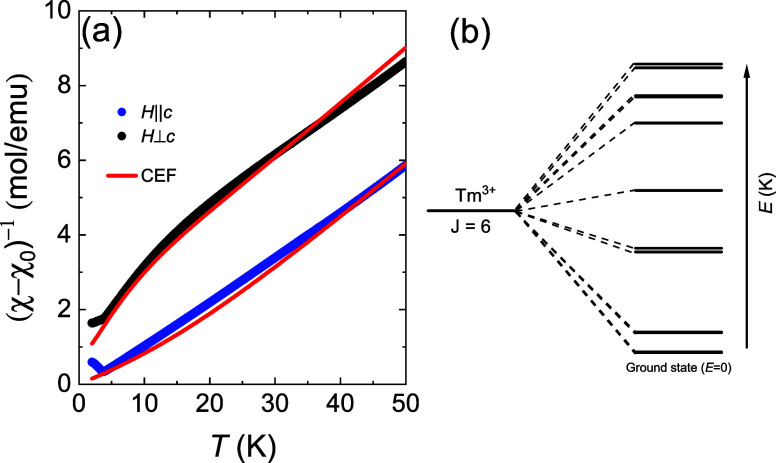
(a) The inverse susceptibility, after subtracting χ_0_, is fitted (red line) using the CEF model for various crystallographic orientations within the temperature range of 2 to 50 K. (b) A schematic diagram describing the energy level splitting due to the CEF effect. The energy levels and corresponding wavevectors are presented in Table 2.

**Table 2:** Estimated Crystal Electric Field Parameters, Energy Levels, and Corresponding Wave Functions for TmGa_2_

*B*_2_^0^ (K)	*B*_2_^2^ (K)	*B*_4_^0^ (mK)	*B*_4_^2^ (K)	*B*_4_^4^ (K)	*B*_6_^0^ (μK)	*B*_6_^2^ (mK)	*B*_6_^4^ (μK)	*B*_6_^6^ (μK)	λ_*i*_ (mol/emu)
CEF Parameters									
–0.418	–0.331	0.21	–0.0147	0.0032	2.3	0.17	10	–14	λ_*z*_ = −0.075; λ_*x*,*y*_ = −0.090

*E* (K)	|+6⟩	|+5⟩	|+4⟩	|+3⟩	|+2⟩	|+1⟩	|0⟩	|-1⟩	|-2⟩	|-3⟩	|-4⟩	|-5⟩	|-6⟩
Energy Levels and Wave Functions													
61.04	–0.071	0	0.283	0	–0.554	0	–0.465	0	–0.554	0	0.283	0	–0.071
60.25	0	–0.111	0	0.192	0	–0.671	0	–0.671	0	0.192	0	–0.111	0
54.35	0.111	0	–0.405	0	0.569	0	0	0	–0.569	0	0.405	0	–0.111
54.09	0	0.311	0	–0.555	0	0.308	0	–0.308	0	0.555	0	–0.311	0
48.61	0.091	0	–0.304	0	0.191	0	–0.852	0	0.191	0	–0.304	0	0.091
48.52	0	–0.346	0	0.576	0	0.222	0	0.222	0	0.576	0	–0.346	0
34.30	0	–0.194	0	0.244	0	0.635	0	–0.635	0	0.244	0	0.194	0
22.08	–0.316	0	0.485	0	0.407	0	0	0	–0.407	0	–0.485	0	0.316
21.26	0.323	0	–0.472	0	–0.381	0	0.235	0	–0.381	0	–0.472	0	0.323
4.32	0	–0.605	0	–0.364	0	–0.045	0	0.045	0	0.364	0	0.605	0
4.23	0	0.607	0	0.363	0	0.003	0	0.003	0	0.363	0	0.607	0
0.12	0.623	0	0.318	0	0.104	0	0	0	–0.104	0	–0.318	0	–0.623
0	0.618	0	0.324	0	0.107	0	–0.05	0	0.107	0	0.324	0	0.618

This yield *B*_2_^0^ = −0.44 K, which is close to the estimate from the CEF model fit (−0.418 K), validating the obtained CEF parameters.

When compared to TmCu_2_, the overall crystal electric field acting on Tm^3+^ is approximately 3 times weaker (*B*_2_^0^ = −0.418 compared to −1.16 K in TmCu_2_). This is consistent with longer Tm–Ga bond lengths (average bond length 3.14 Å compared to the average Tm–Cu distance 2.99 Å^65^). Interestingly, the sign of *B*_4_^0^ is flipped in case of TmGa_2_ with respect to TmCu_2_ (0.21 mK vs −1.81 mK in the latter case), while *B*_2_^2^ appears to be enhanced with respect to *B*_2_^0^ (*B*_2_^2^/*B*_2_^0^ = 0.79 in TmGa_2_ and 0.69 in TmCu_2_). This likely results from the fact that in TmGa_2_ the coordination polyhedron around Tm is strongly elongated along the crystallographic *c* direction (see the Figure S5) and overall the variation of bond lengths is higher than in TmCu_2_ (Baur’s distortion index^66^ *d* = 0.039 compared to 0.022 in TmCu_2_). *B*_6_^4^ and *B*_6_^6^ parameters are found to be significantly reduced compared to TmCu_2_, possibly due to stronger screening by conduction electrons.

An important thing to note is that due to the low symmetry and high multiplicity, the correct estimation of CEF parameters (especially in the case of higher-order terms) is difficult and ambiguous when only heat capacity and magnetization are used,^56^ especially in intermetallic systems, where a simple point charge model is drastically insufficient. A detailed CEF analysis would require an analysis of the inelastic neutron scattering data.

Figure 7a depicts the temperature-dependent heat capacity *C*_P_(*T*) of TmGa_2_ and LuGa_2_ measured in the temperature range 2–200 K at constant pressure and zero applied field. Note that the heat capacity of LuGa_2_ was also measured down to 340 mK, but no superconductivity was detected. The sharp anomaly at *T*_N_, as clearly visible in the inset of Figure 7a, confirms the bulk nature of the magnetic phase transition in TmGa_2_. The specific heat jump at the AFM phase transition temperature is 4.8 J/(mol K), which is much lower than the expected value (20.54 J/mol K) of the Tm^3+^ ion as per the mean field theory.^67^ Figure 7b shows the heat capacity data measured at various applied fields, which reveal the evolution of the magnetic transition with the applied magnetic field. The transition temperature shifts to a lower temperature with the increase of the magnetic field, which further confirms the AFM phase transition in TmGa_2_. The temperature dependence of magnetic heat capacity, *C*_mag_, as presented in Figure 7c, has been calculated using the formula *C*_mag_ = *C*_P_(TmGa_2_) – *C*_P_(LuGa_2_). *C*_mag_(*T*) data reveal a broad hump around 15 K, which is attributed to the Schottky anomaly due to CEF splitting. The Schottky heat capacity for a multilevel nondegenerate system can be defined as follows

6 $$\begin{array}{l} C_{Sch}\left(T\right)=\left(\frac{R}{T^{2}}\right)[\sum_{i}e^{-\Delta_{i}/T}\sum_{i}{\Delta_{i}}^{2}e^{-\Delta_{i}/T}\\ -{\left(\sum_{i}\Delta_{i}e^{-\Delta_{i}/T}\right)}^{2}]{\left(\sum_{i}e^{-\Delta_{i}/T}\right)}^{-2} \end{array}$$

where *R* is the molar gas constant and Δ_*i*_ energy gap splitting of the *i*th state.^68^

**Figure 7:**
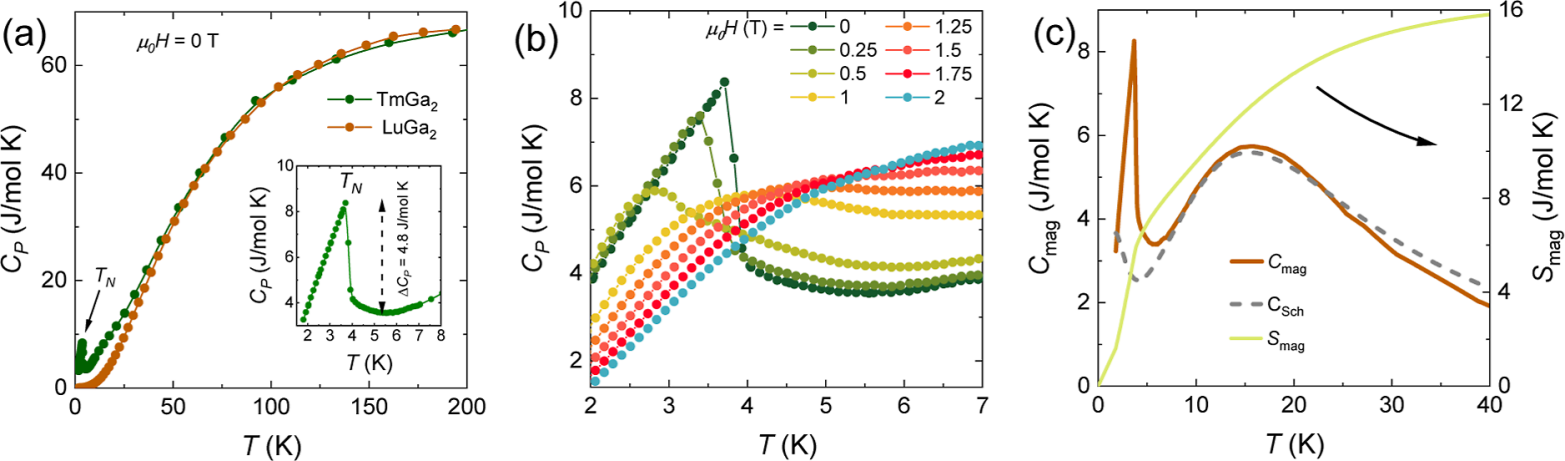
(a) The temperature dependence of heat capacity data of TmGa_2_ and LaGa_2_ in the temperature range 2–200 K in the absence of an external magnetic field. The inset shows a closer view of the heat capacity data of TmGa_2_ near the magnetic ordering temperature. (b) Heat capacity data of TmGa_2_ under various magnetic fields along the *H*∥*c* direction. (c) The magnetic and calculated Schottky contributions to the heat capacity as a function of temperature. The right axis shows the estimated magnetic entropy. Low-temperature heat capacity of LuGa_2_ is shown in Figure S4.

The Schottky heat capacity calculated using the CEF model is shown with a dashed line in Figure 7c. The right axis of Figure 7c shows the magnetic entropy calculated by integrating *C*_mag_/*T*. At the magnetic ordering temperature *S*_mag_ is 5.8 J/(mol K), much below the expected value *R* ln­(2*J* + 1) = 21.3 J/(mol K), but close to *R* ln(2) = 5.76 J/(mol K), which suggests a quasi-doublet ground state resulting from the orthorhombic CEF splitting, similar as in the case of TmCu_2_.^56^ The total amount of entropy recovered up to 40 K (above the Schottky hump centered at ca. 15 K) amounts to approximately 75% of the expected value, highlighting the contribution of higher crystal field levels (see Table 2).

A magnetic phase diagram is constructed utilizing a contour plot of heat capacity data, phase transition temperatures, and critical fields obtained from magnetization data along *H*∥*c*, as shown in Figure 8. The overall phase diagram can be divided into three separate magnetic states: paramagnet (PM), antiferromagnet (AFM), and metamagnet (MM). Below 4 K, TmGa_2_ goes through an antiferromagnetic phase transition. As expected, the AFM transition moves to lower temperatures with the increase of magnetic field. At 2 K, a metamagnetic phase is seen within the AFM phase, with a critical field *H*_c_ = 0.15 T. The *H*_*c*_ decreases with increasing temperature, forming a separate region.

**Figure 8:**
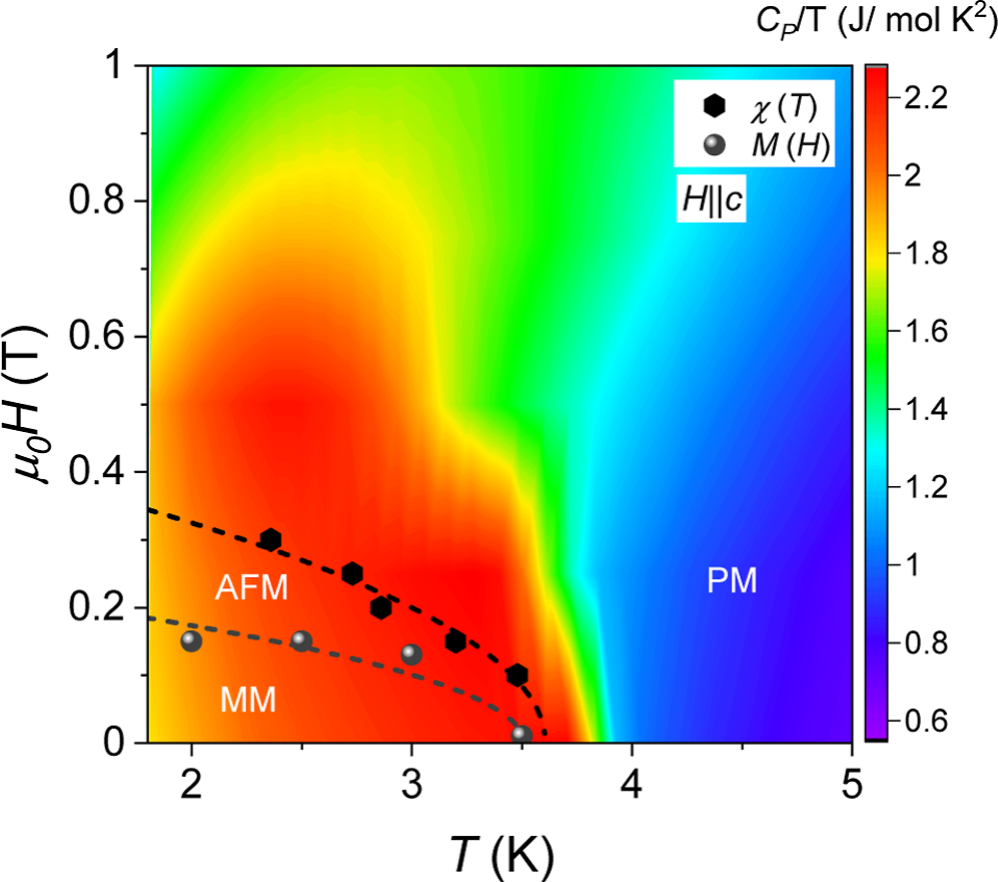
Magnetic phase diagram of TmGa_2_ along *H*∥*c* constructed using magnetic susceptibility, magnetization, and heat capacity data.

## 4 Conclusions

In conclusion, we have performed a systematic investigation of the structural stability and magnetic properties of single-crystal TmGa_2_. Good-quality crystals, which form in an orthorhombic structure, were grown using the flux method, employing Pb as a flux. Ab initio calculations of electronic structure and chemical bonding, together with machine-learning-based chemical pressure analysis, revealed that the reason for the preference for the KHg_2_-type orthorhombic variant observed for only two of the members of the RGa_2_ (R-lanthanides), TmGa_2_ and LuGa_2_, is due to pressure-optimizing transition from the hexagonal AlB_2_-type structure. Consistent with the lanthanide contraction effect, i.e., gradual decrease in atomic and ionic size across the lanthanide series, the chemical pressure acting to compress Ga–Ga bonds within the graphite-like layers increases with the atomic number from La to Lu, reaching a critical value for Tm and Lu, at which the structural phase transition occurs.

The temperature-dependent magnetic susceptibility and heat capacity measurements suggest an antiferromagnetic phase transition at 3.5 K. A metamagnetic transition is observed in the isothermal magnetization data along *H*∥*c*. The magnetic susceptibility further suggests the presence of a crystalline electric field effect in this compound, which has been analyzed using a multilevel energy scheme, indicating the splitting of 13 nondegenerate levels. Estimated energy levels agree well with the observed Schottky anomaly in the heat capacity data. The CEF effect lifts the 2J + 1-fold degeneracy of the Tm^3+^ multiplet, which influences several physical properties, including the magnetic moment and magnetic anisotropy, and results in deviations from Curie–Weiss behavior due to thermal population of excited levels. Additionally, it causes a Schottky anomaly in the heat capacity and allows field-induced transitions by mixing adjacent CEF levels. Further investigation like neutron diffraction measurement is required to reveal the magnetic configuration of antiferromagnetic TmGa_2_.

Overall, our work provides a microscopic understanding of how chemical pressure stabilizes specific structural motifs in lanthanide gallides and uncovers complex metamagnetic behavior in TmGa_2_. These insights could be useful in the design and discovery of new rare-earth intermetallics with tunable structural and magnetic properties.

## Supplementary Material



## Notes

XRD patterns, EDS spectra, temperature-dependent electrical resistivity of TmGa_2_, low-temperature heat capacity of LuGa_2_, relaxed lattice and structure parameters of REGa_2_ (RE—rare earth) compounds, and comparison of crystal structures of TmGa_2_ and TmCu_2_ (PDF)

## Abbreviations

AFM: antiferromagnet
BFGS: Broyden–Fletcher–Goldfarb–Shanno
CEF: crystalline electric field
COBI: crystal orbital bond index
COHP: crystal orbital Hamilton population
DFT: density functional theory
DOS: density of states
EDS: Energy Dispersive (X-ray) Spectroscopy.
FC: field-cooled
ML-CP: machine learning-based chemical pressure
PAW: projector-augmented wave
PBE GGA: Perdew–Burke–Ernzerhof Generalized Gradient Approximation
PPMS: physical property measurement system
PZ LDA: Perdew–Zunger Local Density Approximation
QE: Quantum Espresso
RKKY: Ruderman–Kittel–Kasuya–Yosida
VSM: vibrating sample magnetometer
xc: exchange-correlation
XRD: X-ray diffraction
ZFC: zero field-cooled.
