# Supplementary material for: Study of the Stability of Structural Variants and Magnetic Properties of TmGa2
Source: Inorg Chem. 2026 May 22;65(22):12153–62. doi: 10.1021/acs.inorgchem.5c06073 (PMC13308880; doi:10.1021/acs.inorgchem.5c06073)
Supplement: Supplementary file 1 [file ic5c06073_si_001.pdf]

SUPPLEMENTARY INFORMATION

Study of the stability of structural variants and magnetic properties of $TmGa_2$

Sudip Malick[1, 2,*] Michał J. Winiarski, Hanna Świątek,[1, 2] and Tomasz Klimczuk[1, 2,‡]

[1] *Faculty of Applied Physics and Mathematics, Gdansk University of Technology, Narutowicza 11/12, 80-233 Gdańsk, Poland*

[2] *Advanced Materials Center, Gdansk University of Technology, Narutowicza 11/12, 80-233 Gdańsk, Poland*

∗ sudip.malick@pg.edu.pl † michal.winiarski@pg.edu.pl ‡ tomasz.klimczuk@pg.edu.pl

X-ray diffraction:

The Powder XRD pattern of $LuGa_2$, along with Le Bail refinement, is shown in Fig. S1. The obtained refinement parameters for $TmGa_2$ and $LuGa_2$ are presented in Table S1.

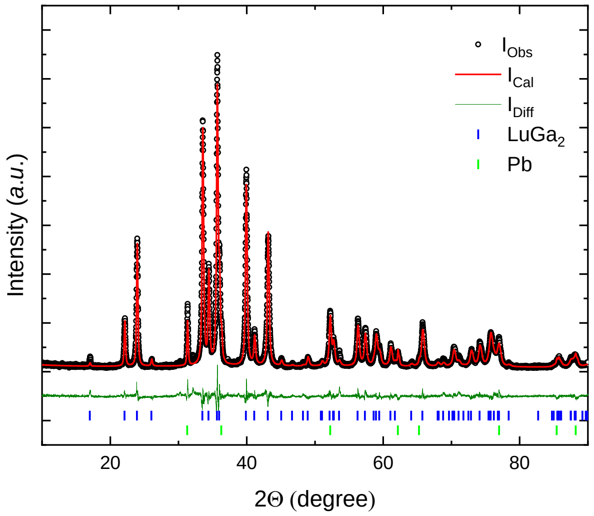

Fig S1. The powder X-ray diffraction pattern of the crushed $LuGa_2$ single crystals, shown as black dots. The calculated pattern (Le Bail) is shown by the red line. The Bragg positions are represented by the blue ($LuGa_2$) and green (Pb) vertical lines. The difference between the observed and the estimated intensity is represented by the oval line.

Table S1. XRD refinement parameters of $TmGa_2$ and $LuGa_2$.

	**$TmGa_2$**	**Pb**	**$LuGa_2$**	**Pb**
Crystal structure	Orthorhombic	Cubic	Orthorhombic	Cubic
Space group	*Imma*	*Fm-3m*	*Imma*	*Fm-3m*
a (Å)	4.2121(2)	4.9519(8)	4.1957(1)	4.9499(2)
b (Å)	6.8924(3)		6.8424(2)	
c (Å)	8.0679(3)		8.0424(2)	
V (Å^3)	234.222(2)	121.43(6)	230.891(1)	121.28(1)
R_{wp} (%)	7.09		9.88	
χ^2	1.45		1.73	

**$TmGa_2$**				
Atom	*Site*	*x*	*y*	*z*
Tm	4e	0	1/4	0.5567(2)
Ga	8i	0.0511(2)	1/4	0.1612(2)
**LuGa2**				
Lu	4e	0	1/4	0.5569(2)
Ga	8i	0	0.06	0.1590(2)

**Pb**				
Atom	*Site*	*x*	*y*	*z*
Pb	4a	0	0	0

Energy Dispersive Spectroscopy:

The EDS data of $TmGa_2$ and $LuGa_2$ are shown in Fig. S2 (a) and (b), respectively. Backscattered electron images show a single-color contrast, suggesting homogeneity and a single phase in both compounds, as shown in Fig. S2 (c) and (d).

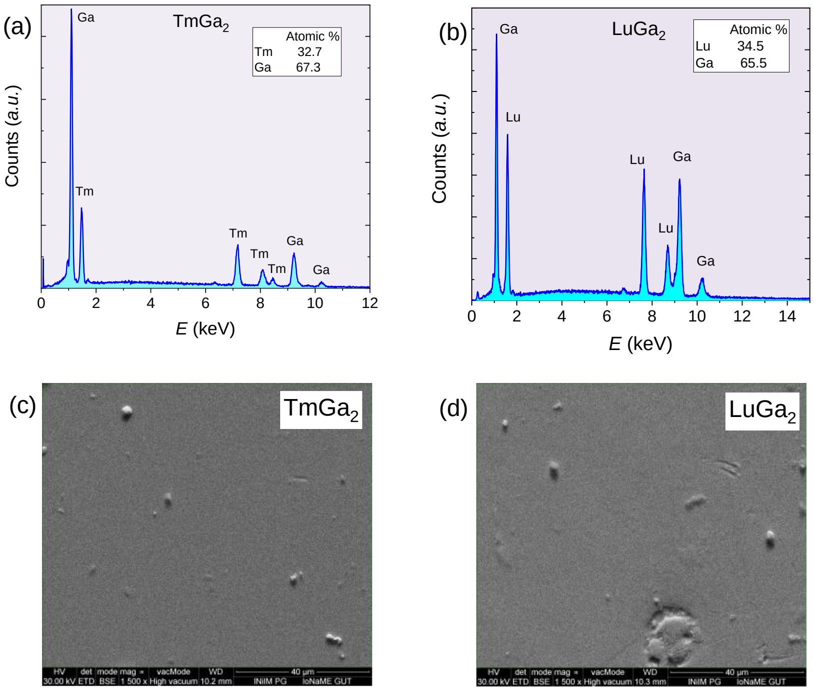

Fig. S2. The EDS data of (a)$TmGa_2$ and (b) $LuGa_2$, corresponding backscattered electron images are shown in (c) and (d), respectively.

Electrical resistivity:

The temperature-dependent electrical resistivity of $TmGa_2$ is shown in Fig. It exhibits metallic behavior over the temperature range 1.8-300 K, with an anomaly due to the antiferromagnetic transition. The estimated resistivity ratio (RRR) is about 10, suggesting a good-quality grown crystal.

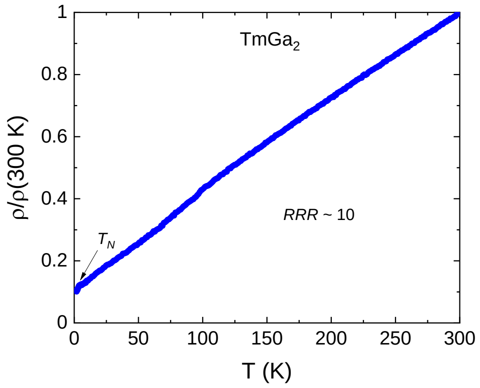

Fig. S3. Normalized electrical resistivity of $TmGa_2$ measured in the temperature range 1.8-300 K.

Low-temperature heat capacity of $LuGa_2$:

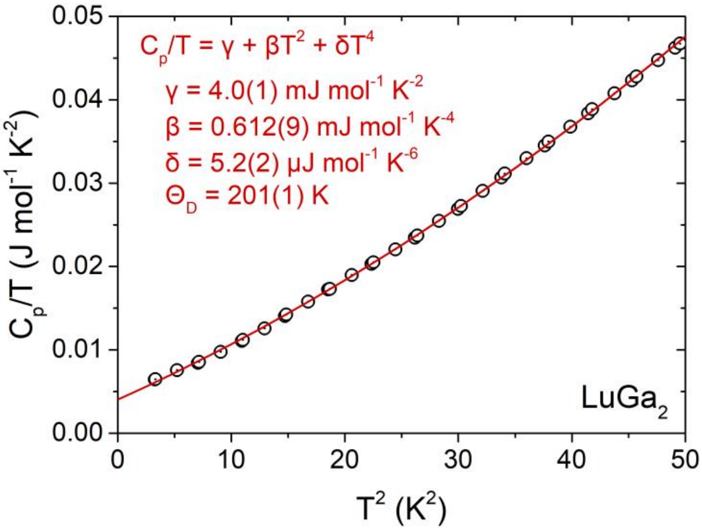

Fig. S4. Fit (red line) to the low temperature heat capacity of $LuGa_2$.

Ab initio calculations:

Table S2. Unit cell parameters of hexagonal (AlB_2-type, space group #191, R atoms at the 1a site, Ga at 2d) RGa_2 compounds relaxed using PBE GGA xc functional.

**Compound:**	***a* (Å)**	***c* (Å)**
$HoGa_2$	4.19600	4.07806
$ErGa_2$	4.18853	4.05831
$TmGa_2$	4.18146	4.03587
$LuGa_2$	4.17156	3.98308

Table S3. Unit cell and structural parameters of orthorhombic (KHg_2-type) $TmGa_2$ relaxed using PBE GGA xc functional.

**Space group:**		I m m a (#74)		
**Unit cell parameters:**				
a (Å)		6.88556		
b (Å)		4.20570		
c (Å)		8.17632		
**Structure parameters:**				
Atom	*Site*	*x*	*y*	*z*
Tm	4e	0	¼	0.18850
Ga	8i	0.30119	¼	0.90956

Table S4. Unit cell parameters of hexagonal (AlB_2-type, space group #191, R atoms at the 1a site, Ga at 2d) RGa_2 compounds relaxed using PZ LDA xc functional.

**Compound:**	***a* (Å)**	***c* (Å)**
YGa_2	4.11358	4.00622
$GdGa_2$	4.12808	4.00552
$TbGa_2$	4.11793	3.97773
$DyGa_2$	4.10857	3.95169
$HoGa_2$	4.10064	3.92589
$ErGa_2$	4.09521	3.89695
$TmGa_2$	4.09180	3.86325
$LuGa_2$	4.08077	3.81414

Table S5 Unit cell parameters of hexagonal (AlB_2-type, space group #191) YGa_2 relaxed using PZ LDA xc functional using the Hartwigesen-Goedecker-Hutter pseudopotential.

**Compound:**	***a* (Å)**	***c* (Å)**
YGa_2	4.03192	3.94048

Comparison of $TmCu_2$ and $TmGa_2$ crystal structure

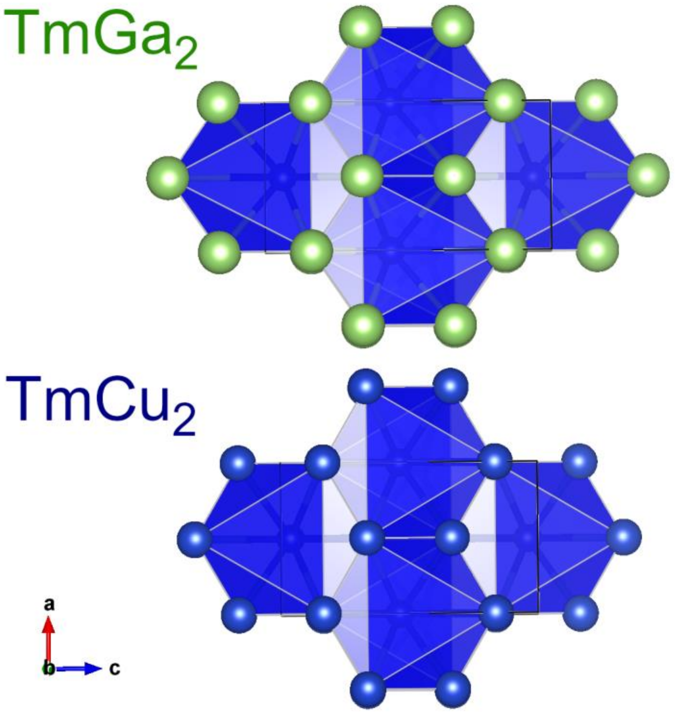

Fig. S5. $TmGa_2$ (top) and $TmCu_2$ (bottom) crystal structures viewed along the crystallographic b *axis. Note the elongation of the Tm-Ga coordination polyhedra along the* c *direction.*
